# Dysbiosis and Links of the Middle Ear, Nasal, and Oral Microbiota in Chronic Otitis Media with Effusion

**DOI:** 10.1002/mco2.70550

**Published:** 2025-12-14

**Authors:** Jin Li, Shenglong Xu, Qin Gu, Xuan Sun, Yahan Zhao, Peng Zhang, Yi Li, Yan Zhao, Luo Zhang

**Affiliations:** ^1^ Department of Otolaryngology Head and Neck Surgery Beijing TongRen Hospital Capital Medical University Beijing China; ^2^ Beijing Institute of Otolaryngology Beijing Laboratory of Allergic Diseases Beijing Key Laboratory of New Medicine and Diagnostic Technology Research for Nasal Disease Beijing China; ^3^ Beijing Key Laboratory For Genetics of Birth Defects Beijing Pediatric Research Institute MOE Key Laboratory of Major Diseases in Children Rare Disease Center Beijing Children's Hospital Capital Medical University National Center for Children's Health Beijing China; ^4^ Department of Allergy Beijing TongRen Hospital Capital Medical University Beijing China

**Keywords:** chronic otitis media with effusion, microbial dysbiosis, middle ear microbiota, nasal microbiota, oral microbiota, 16S rRNA sequencing

## Abstract

Chronic otitis media with effusion (COME) is a common condition that can lead to hearing loss without acute inflammation. However, its underlying pathogenesis remains poorly understood, particularly with regard to microbial contributions. In this study, we characterized and compared the microbiota of the middle ear, nasal cavity, and oral cavity in 100 patients with COME and 77 controls using 16S rRNA gene sequencing. Alpha diversity, assessed by the Pielou index, was significantly reduced in the middle ear microbiota of COME patients compared with controls, and microbial compositions differed markedly across all sampled sites. Notably, *Aeromonas*, *Serratia*, and *Lactococcus* were enriched in both middle ear and nasal samples from COME patients. Among these, *Aeromonas* achieved the highest predictive value for COME in otic samples, whereas *Lactococcus* showed the strongest performance in nasal samples, with strong inter‐site correlations. Functional analysis revealed the enrichment of pathways related to biofilm formation and depletion of antibiotic biosynthesis in COME‐associated microbiota. These findings highlight the presence of microbial dysbiosis, particularly the interplay between nasal and middle ear microbiota, as a potential contributor to the COME pathogenesis, and suggest novel microbial targets for diagnosis and therapeutic intervention.

## Graphical abstract

This study profiled the microbiota of the middle ear, nasal cavity, and oral cavity in 100 COME patients and 77 controls using 16S rRNA sequencing. Key COME‐associated taxa were enriched in both otic and nasal sites, with strong inter‐site correlations. These findings highlight nasal‐otic microbial interplay in COME pathogenesis and provide potential targets for diagnosis and intervention.

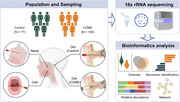

## 1 Introduction

Chronic otitis media with effusion (COME) is characterized by persistent fluid accumulation in the middle ear cavity for over three months without symptoms or signs of acute inflammation. It can cause significant hearing loss, impacting language development in children and quality of life in adults. OME affects approximately 20% of children and 0.6% of adults [1], with 5%–10% progressing to COME [2]. While acute OME often resolves spontaneously, COME persists for extended periods and typically requires tympanostomy tube placement.

Multiple factors contribute to the pathogenesis of COME, including eustachian tube dysfunction, persistent inflammation, and mucosal remodeling. In recent years, the microbiome has been recognized to play a crucial role in the pathogenesis of COME [3], and the upper respiratory tract (URT) is essential for maintaining middle ear health [4]. The URT microbiota normally maintains an ecological balance that supports key functions, such as antagonizing pathogens and modulating immunity [5]. However, microbial dysbiosis—where the microbial community shifts from a physiological to a pathological state—can disrupt this balance, potentially leading to URT inflammation and contributing to the development of COME [6]. Common pathogens causing otitis media, such as *Haemophilus influenzae* and *Streptococcus pneumoniae*, are generally thought to ascend from the URT into the middle ear via the eustachian tube [7]. These bacteria are commensals of the oral and nasal cavity, and their presence in the middle ear is often linked to microbial dysbiosis.

In addition to the traditional otopathogens, recent studies using next‐generation sequencing of middle ear effusions in COME have identified unculturable microorganisms [8, 9, 10, 11]. The advent of 16S rRNA gene sequencing has revealed a much greater microbial diversity in the middle ear than previously recognized by culture‐based methods. These include low‐abundance taxa and anaerobic species that may modulate inflammation through interbacterial interactions or metabolic activity. Despite these advancements, few studies have compared the microbiota composition in the middle ear under normal and COME conditions, complicating the understanding of the pathogenesis. Furthermore, previous research has primarily focused on the microbiota of the middle ear, thus limiting the insight into the role of the URT [12, 13].

To better address these issues, we collected microbial samples from middle ear effusions, normal middle ear swabs, as well as nasal and oral samples from COME patients and controls. We aim to investigate the role of the microbiome in pathogenesis by analyzing the microbiota composition and differences across the middle ear, nasal cavity, and oral cavity in both groups and demonstrating the relationships between the microbiota of the middle ear and the URT.

By integrating 16S rRNA gene sequencing with ecological, biomarker, network, and functional analyses across the middle ear, nasal cavity, and oral cavity, this study reveals microbial alterations associated with COME and underscores the close microbial relationship between the middle ear and the URT. Profiling multiple anatomical niches in a large cohort provides new evidence that the URT may contribute to the microbial shifts observed in COME. These findings highlight potential microbial signatures and functional changes relevant to disease development and offer a foundation for future work aimed at improving early identification and exploring targeted antimicrobial or microbiome‐based therapeutic strategies.

## 2 Results

### 2.1 Study Population and Baseline Characteristics

**FIGURE 1:**
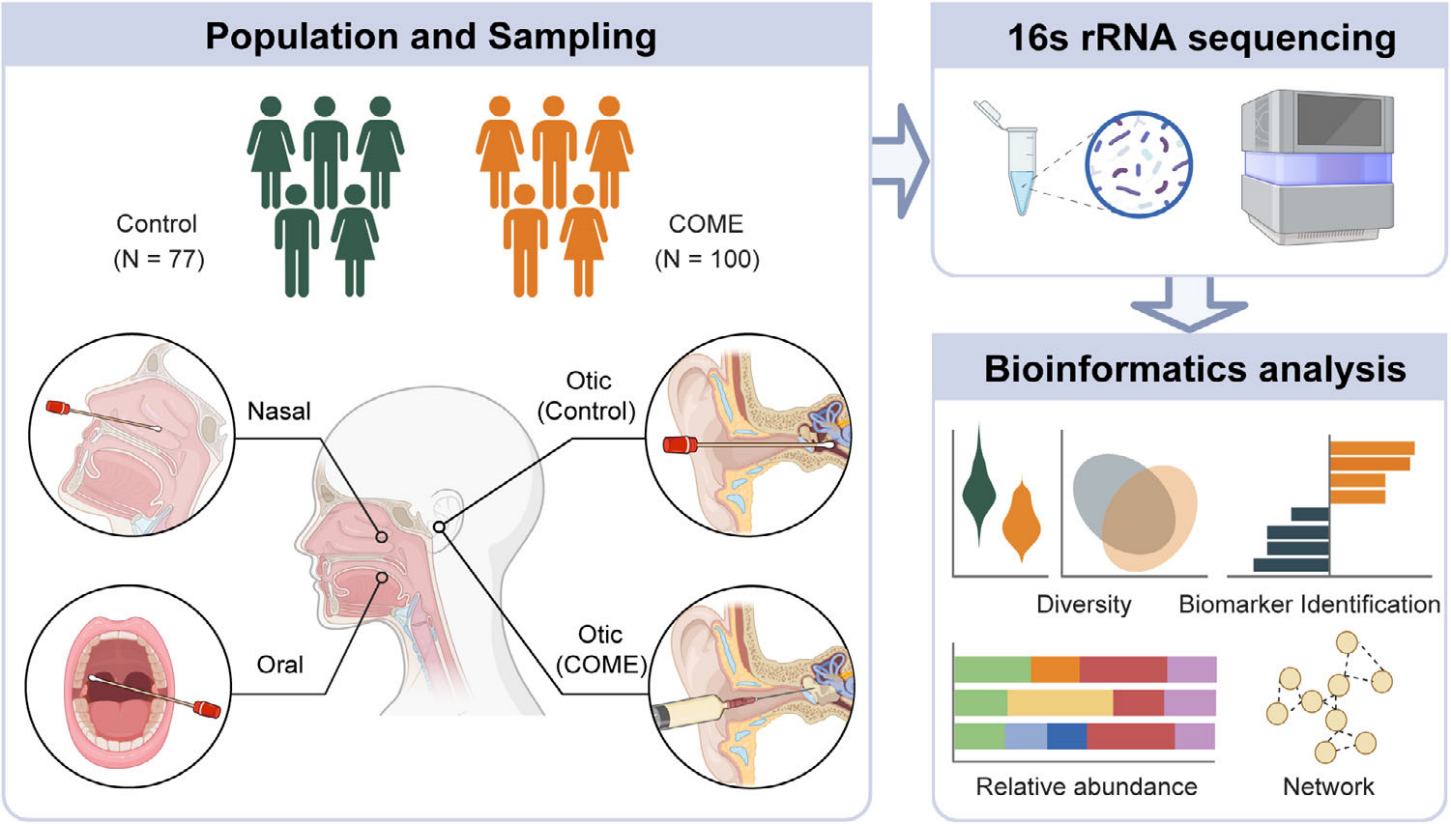
Participants and study design. COME, chronic otitis media with effusion; LDA, linear discriminant analysis.

A total of 177 participants were enrolled in this study, including 100 patients with COME and 77 controls. The study design is illustrated in Figure 1. Among the 77 controls, 25 individuals underwent middle ear surgery, during which normal middle ear samples were collected. The surgical indications included otosclerosis (15 cases, 60%), middle ear malformation (5 cases, 20%), traumatic ossicular dislocations (4 cases, 16%), and acoustic neuroma (1 case, 4%). The baseline characteristics of the two groups are presented in Table 1. Compared with the Control group, the COME group had an older age (*p* < 0.001) and a higher prevalence of chronic rhinosinusitis (CRS, *p* < 0.001) and laryngopharyngeal reflux disease (LPRD, *p* = 0.02). No significant differences were observed between the groups in terms of sex composition, prevalence of allergic rhinitis (AR), history of smoking or alcohol consumption, snoring, or history of COVID‐19. Among the patients with COME, 83 participants completed pure tone audiometry, with a median [interquartile range (IQR)] air‐bone gap of 25 dB. A total of 55 patients completed an acoustic impedance test, of whom 35 (63.6%) exhibited type C tympanograms, 19 (34.5%) exhibited type B tympanograms, and 1 (1.8%) exhibited a type A tympanogram.

**TABLE 1:** Participants’ characteristics.

	COME group (*N* = 100)	Control group (*N* = 77)	*p*‐value
Age (years)	50 [32, 63]	33 [24, 44]	**<0.001**
Female (%)	39 (39.0)	38 (49.4)	0.173
Allergic rhinitis (%)	20 (20.0)	7 (9.1)	0.058
Chronic rhinosinusitis (%)	38 (38.0)	6 (7.8)	**<0.001**
Laryngopharyngeal reflux disease (%)	21 (21.0)	6 (7.8)	**0.020**
Snoring (%)	33 (33.0)	19 (24.7)	0.248
COVID‐19 history (%)	27 (27.0)	25 (32.5)	0.506
Alcohol consumption history (%)	29 (29.0)	24 (31.2)	0.869
Tobacco consumption history (%)	21 (21.0)	15 (19.5)	0.852
Disease duration (months)	12 [3, 44]	NA	NA
Airi‐bone gap (dB)	25 [17, 34]	NA	NA
Acoustic impedance (%)			NA
As	1 (1.8)	NA	
B	19 (34.5)	NA	
C	35 (63.6)	NA	
Otic sampling side (%)			0.153
Left	37 (37.0)	12 (54.5)	
Right	63 (63.0)	10 (45.5)	

*Note*: Categorical variables are presented as *N* (%), and continuous variables are presented as median [IQR].

Abbreviations: COME, chronic otitis media with effusion; NA, not applicable.

### 2.2 Microbial Diversity and Composition across Otic, Nasal, and Oral Samples

**FIGURE 2:**
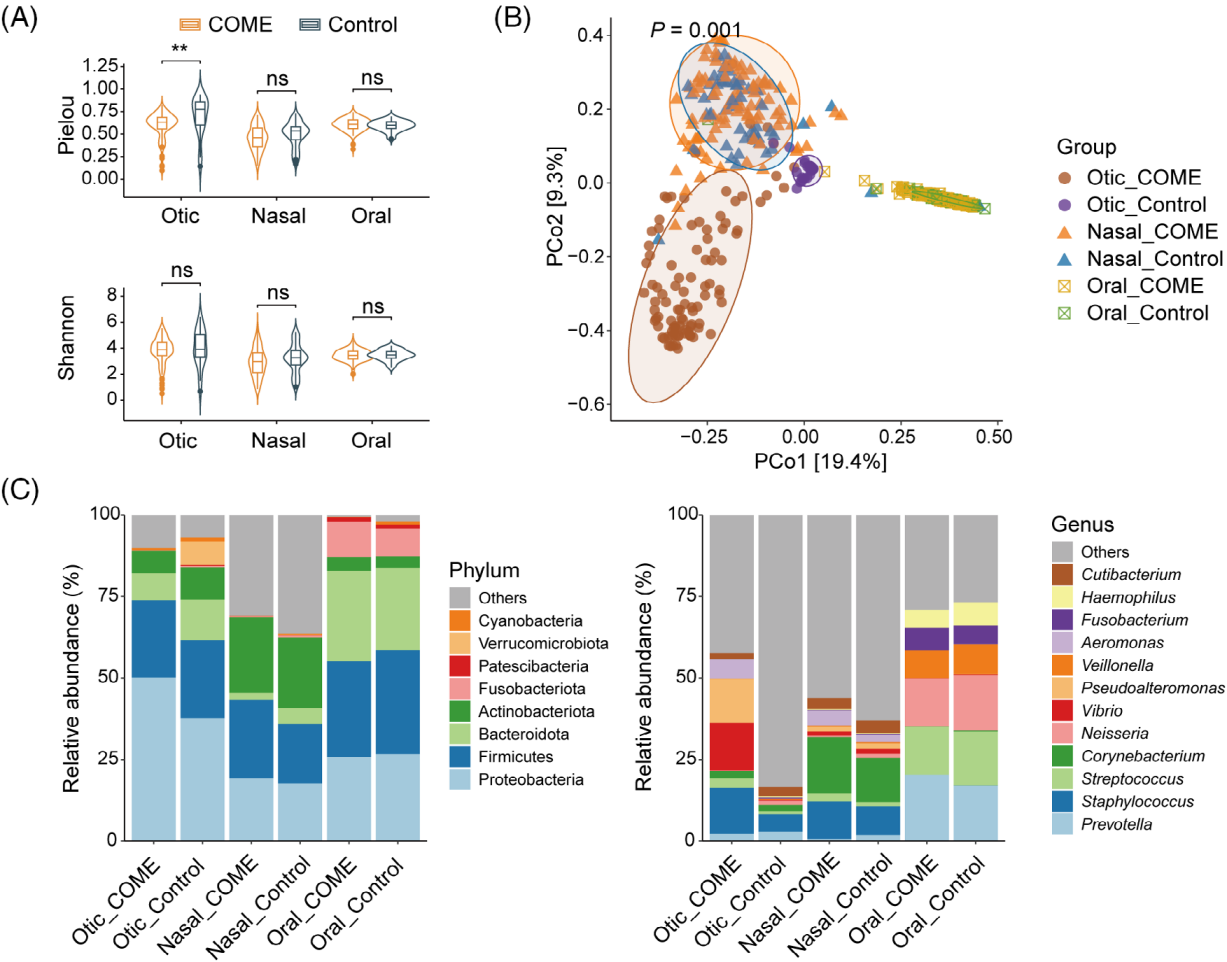
Diversity and taxonomic composition in COME and Control groups across otic, nasal, and oral samples. (A) Comparison of alpha diversity (Pielou and Shannon indices) between COME and Control groups across sites. (B) Principal coordinate analysis based on Bray–Curtis distance. (C) Mean relative abundance of the top 8 phyla and the top 12 genera of the sample groups. COME, chronic otitis media with effusion. The analysis included *n* = 100 (Otic_COME), *n* = 25 (Otic_Control), *n* = 96 (Nasal_COME), *n* = 77 (Nasal_Control), *n* = 100 (Oral_COME), and *n* = 76 (Oral_Control) samples. *p < 0.05; **p < 0.01; ***p < 0.001.

A total of 100 tympanic effusion samples from patients and 25 normal otic samples from controls, 96 nasal samples from patients and 77 from controls, and 100 oral samples from patients and 76 from controls were included in the analysis after filtering out unqualified samples. Regarding alpha diversity, the COME group in otic samples demonstrated a significantly lower Pielou index compared with the controls (*p* = 0.002; Figure 2A), while no difference was observed in the Shannon index. Similarly, no significant differences in either Pielou or Shannon indices were found between the groups in nasal or oral samples. Analysis of the Simpson and Chao1 indices revealed no significant differences between groups in otic or nasal samples, except for a decreased Chao1 index in the oral microbiota of COME patients (*p* = 0.006; Figure S1A). The principal coordinate analysis (PCoA) plot based on Bray–Curtis distances illustrates the beta diversity of microbial communities across the groups (Figure 2B). Distinct clustering patterns were observed among otic, nasal, and oral samples from the COME and the Control groups, indicating significant differences in microbial composition (permutational multivariate analysis of variance, perMANOVA, *p* = 0.001). Notably, normal middle ear samples clustered closer to nasal samples than to oral samples, suggesting a microbial composition more similar to the nasal microbiota. In contrast, COME middle ear samples formed a distinct cluster, highlighting a unique microbial community structure. Furthermore, significant differences in beta diversity between the COME and Control groups were observed at each site (Figure S1B).

At the phylum level, proteobacteria and firmicutes were the dominant taxa across most groups, with proteobacteria being particularly abundant in otic samples, while firmicutes were more prevalent in oral samples (Figure 2C). The phylum‐level compositions for the three sample sites, stratified by disease state, are detailed in Figure S2A. At the genus level, distinct differences were observed across sample types and disease states. In otic samples, *Staphylococcus*, *Vibrio*, *Pseudoalteromonas*, and *Aeromonas* were more abundant in the COME group than in the Control group, suggesting a potential microbial shift associated with the disease. *Staphylococcus* and *Corynebacterium* were enriched in nasal samples, while *Prevotella* and *Streptococcus* were predominant in oral samples. The genus‐level compositions for the three sites are shown in Figure S2B, respectively. The majority of genera were distributed across all three sites, regardless of COME status, highlighting a shared core microbiome among otic, nasal, and oral niches (Figure S2C).

### 2.3 Key Microbial Biomarkers Shared between Otic and Nasal Sites in the COME Group

**FIGURE 3:**
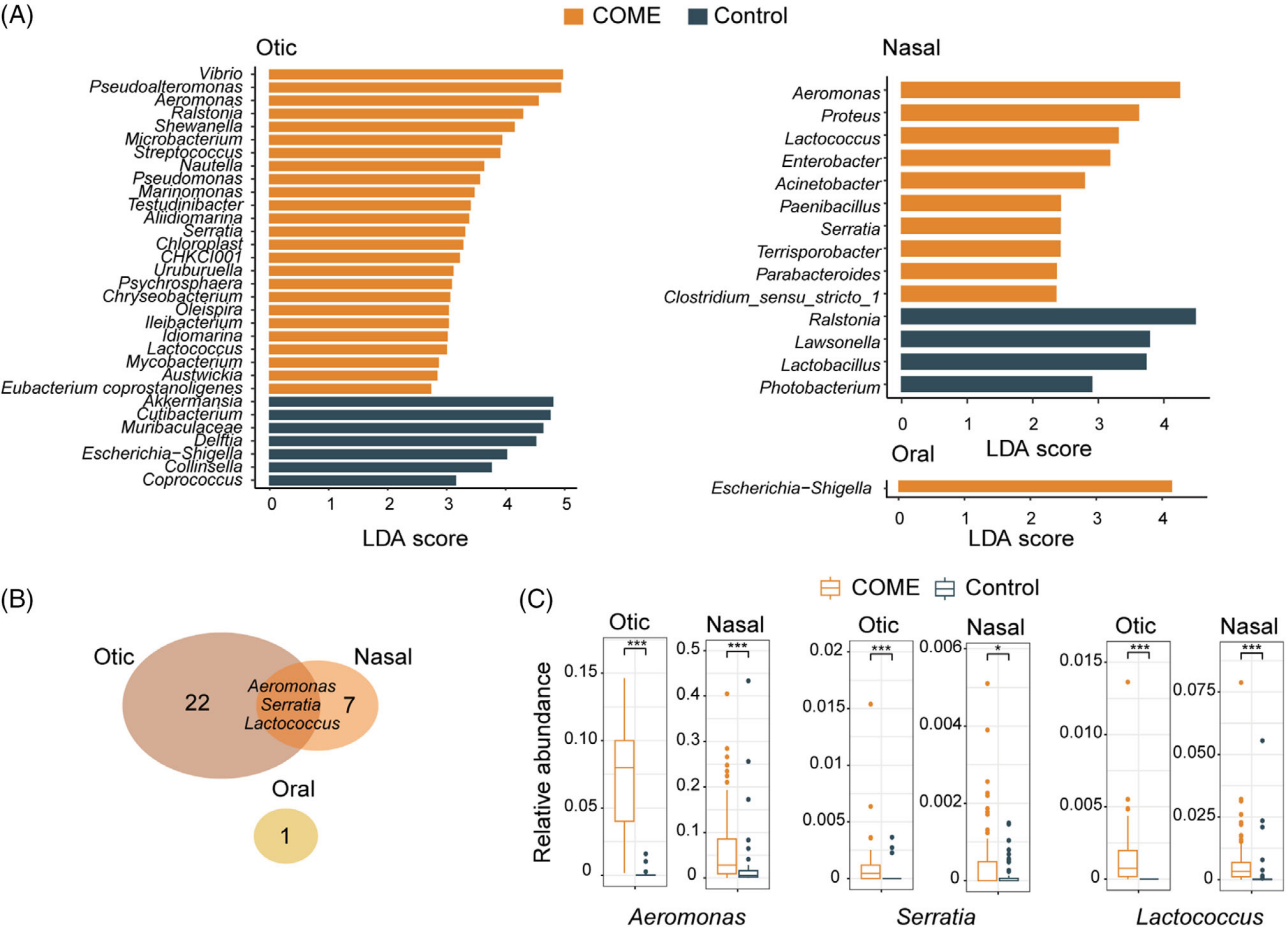
Biomarkers of COME and Control groups. (A) Biomarkers identified across different sites based on the linear discriminant analysis effect size algorithm. (B) Venn diagram showing biomarkers shared within the COME group. (C) Relative abundances of key biomarkers across sites. LDA, linear discriminant analysis; COME, chronic otitis media with effusion. The boxplot shows the distribution of relative abundance, with the box representing the interquartile range (IQR), the vertical line within the box indicating the median, and the whiskers extending to the maximum and minimum values within the range of Q3 + 1.5 × IQR and Q1 − 1.5 × IQR. Outliers are marked as individual points. The analysis included *n* = 100 (Otic_COME), 25 (Otic_Conotrl), 96 (Nasal_COME), 77 (Nasal_Control), 100 (Oral_COME), and 76 (Oral_Contro) samples. *p < 0.05; **p < 0.01; ***p < 0.001.

Microbial biomarkers identified using the linear discriminant analysis (LDA) effect size (LEfSe) algorithm in otic, nasal, and oral samples for the COME and Control groups are presented in Figure 3A. In otic samples, 25 genera were enriched in the COME group, compared with 7 genera that were more abundant in the Control group. Similarly, in nasal samples, 10 genera were enriched in the COME group, while 4 genera were enriched in the Control group. Only a single oral biomarker was identified for the COME group. Notably, *Aeromonas*, *Serratia*, and *Lactococcus* were shared between otic and nasal samples in the COME group (Figure 3B), with no shared genera identified between sites in the Control group.

**FIGURE 4:**
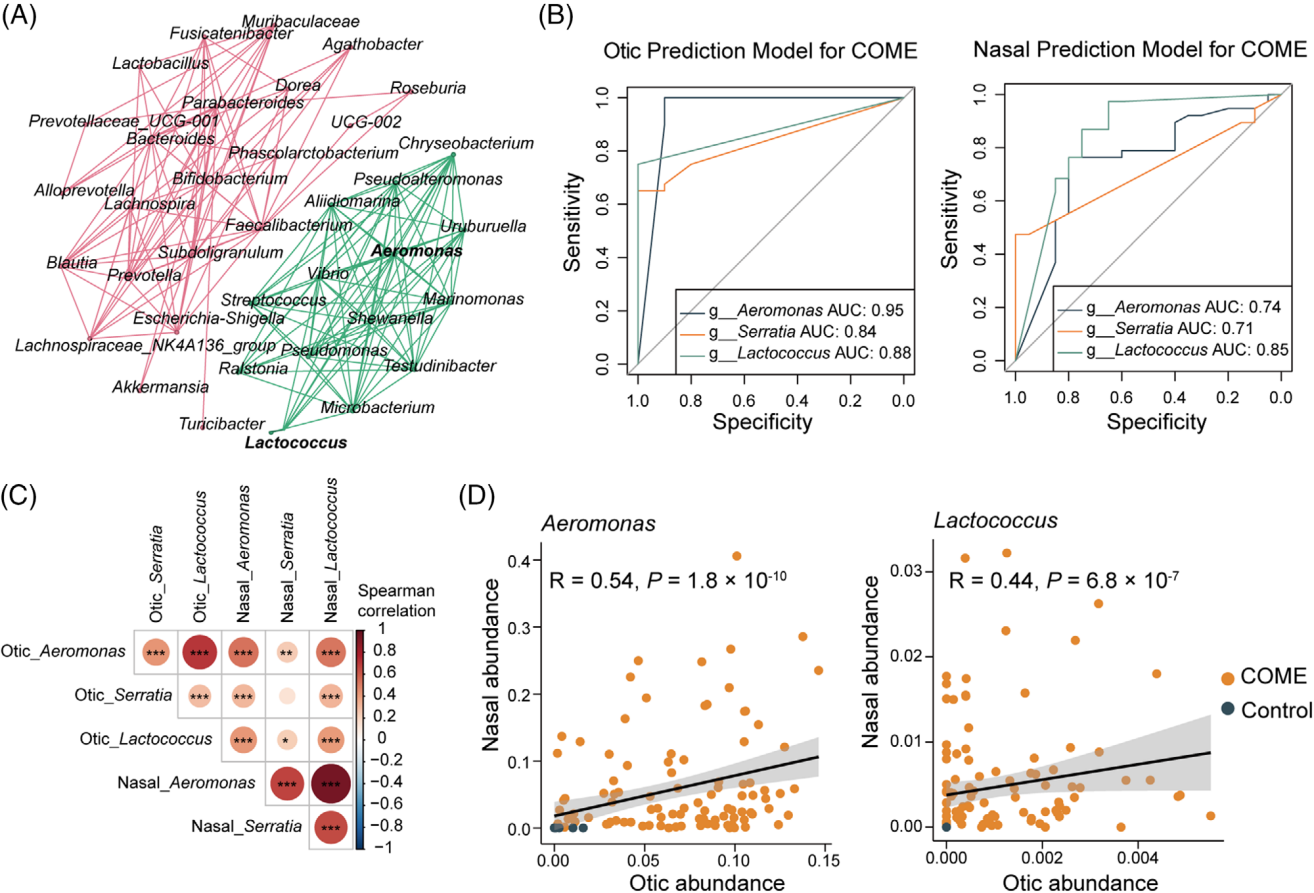
Microbial correlations and predictive modeling in COME. (A) Co‐occurrence network of genera in otic samples from COME patients. (B) ROC curves for the otic and nasal prediction models based on COME key biomarkers. (C) Spearman correlation heatmap of key biomarkers between otic and nasal samples. (D) Scatter plot showing the correlation of *Aeromonas* and *Lactococcus* between nasal and otic samples. The analysis included *n* = 100 (Otic_COME), 25 (Otic_Control), 96 (Nasal_COME), and 77 (Nasal_Control) samples. AUC, area under the curve.

The relative abundances of these three key microbial biomarkers were significantly elevated in the COME group (Figure 3C). Subgroup analysis within the COME group revealed no significant differences in the relative abundances of these genera across subgroups defined by AR, CRS, LPRD, tobacco or alcohol consumption, snoring, or COVID‐19 history (Table S1). Network analysis further revealed that otic genera in the COME samples were clustered into two distinct modules, with microbial biomarkers for COME and Control groups segregating into separate clusters (Figure 4A). *Aeromonas* and *Lactococcus* emerged as central hubs within the same module, highlighting their roles in shaping the microbial communities associated with COME.

Among the three key biomarkers, *Lactococcus* demonstrated strong predictive value for COME (Figure 4B), achieving a high area under the curve (AUC) in otic samples (0.875) and solid performance in nasal samples (0.847). In comparison, *Aeromonas* and *Serratia* showed higher AUCs in otic samples (0.945 and 0.836, respectively) but performed less effectively in nasal samples (0.743 and 0.713, respectively).

Strong positive correlations were observed among the three genera within and across otic and nasal samples, except for the nonsignificant correlation between *Serratia* in otic and nasal samples (Figure 4C). *Aeromonas* exhibited a significant correlation between the middle ear and nasal cavity, with a Spearman's coefficient of 0.54 (*p* < 0.001), while *Lactococcus* also showed a strong correlation between the two sites, with a coefficient of 0.44 (*p* < 0.001) (Figure 4D).

### 2.4 Functional Composition and Differences in COME and Control Groups

**FIGURE 5:**
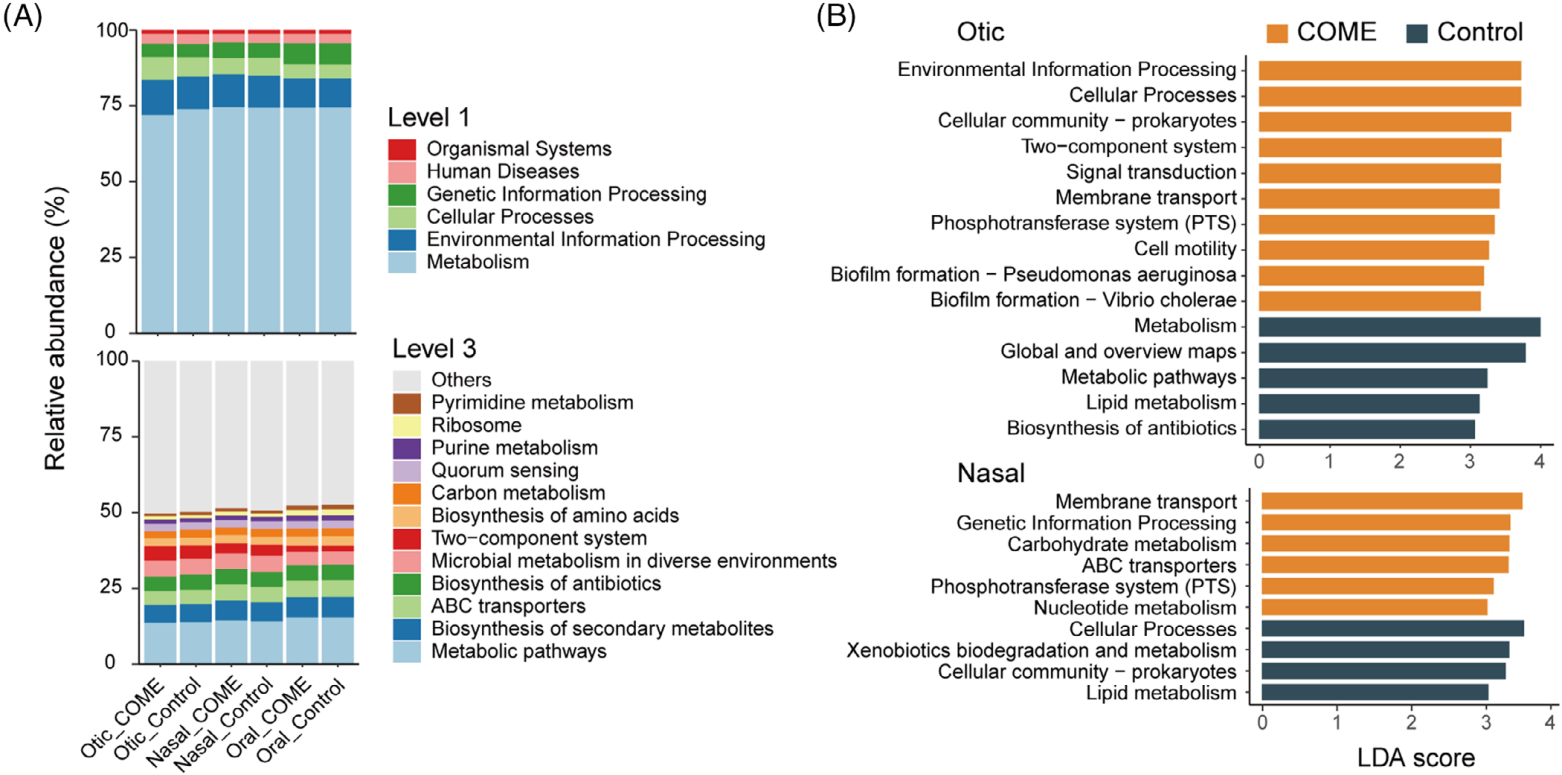
Functional composition prediction of COME and Control groups. (A) Predicted functional composition (Level 1 and Level 3). (B) Functional biomarkers in otic and nasal samples based on the linear discriminant analysis effect size algorithm. COME, chronic otitis media with effusion; LDA, linear discriminant analysis. The analysis included *n* = 100 (Otic_COME), 25 (Otic_Control), 96 (Nasal_COME), 77 (Nasal_Control), 100 (Oral_COME), and 76 (Oral_Control) samples.

Metabolic pathways were the most dominant function at Level 1, followed by environmental information processing and cellular processes, with consistent patterns across otic, nasal, and oral samples in both COME and Control groups (Figure 5A). At Level 3, metabolic pathways, biosynthesis of secondary metabolites, and ATP‐binding cassette (ABC) transporters were the most abundant functions (Figure 5A). Subtle differences in functional composition were observed between the COME and Control groups, particularly in otic and nasal samples.

In otic samples, 10 pathways were upregulated in the COME group, including environmental information processing, cellular processes, cellular community, two‐component system, signal transduction, membrane transport, phosphotransferase system, cell motility, and biofilm formation, while five pathways were upregulated in the Control group, such as metabolism and biosynthesis of antibiotics (Figure 5B). In nasal samples, six pathways were upregulated in the COME group, including membrane transport, genetic information processing, and carbohydrate metabolism, compared with four pathways in the Control group, such as cellular processes, xenobiotic biodegradation, and metabolism (Figure 5B). No significant pathways were identified in oral samples.

## 3 Discussion

COME is highly prevalent and can lead to hearing loss, which negatively impacts language development in children and significantly affects the quality of life in adults. However, its precise pathogenesis remains incompletely understood. Microbial factors are believed to play a crucial role in the development of this condition. In the present study, we employed 16S rRNA sequencing to analyze microbiota from middle ear effusions, normal middle ear swabs, as well as nasal and oral swab samples obtained from both COME patients and controls. Our results identified potential microbial biomarkers associated with COME and highlighted the relationship between middle ear and URT microbiota. To the best of our knowledge, this study represents the largest investigation to date that includes microbiota samples from multiple ecological niches in the context of COME.

COME is a complex, multifactorial condition influenced by a range of etiological factors, including eustachian tube dysfunction, bacterial and viral infections, LPRD, adenoid hypertrophy, snoring, allergic inflammation, smoking, and alcohol consumption. In our study, we observed that the prevalence of AR, CRS, and LPRD was higher in the COME cohort compared with controls. Previous studies have established associations between COME with AR [14, 15, 16], CRS [17, 18], and LPRD [19, 20], suggesting a potential role of the URT in the pathogenesis of COME. However, the direct causal relationships between these conditions remain inconclusive [20, 21]. Despite targeted therapeutic interventions addressing these risk factors, the progression of the disease often persists, suggesting the involvement of additional mechanisms.

Bacterial involvement in COME has been widely documented, with prior research primarily focusing on the analysis of effusions from COME patients [4, 12, 22]. However, these studies often lack comparisons with healthy controls, involve small sample sizes, and fail to examine the distribution and correlation of microbiota in the middle ear, nasal cavity, and oral cavity. To address these gaps, our research utilizes 16S rRNA sequencing to compare the microbial composition and differences across multiple ecological niches in both COME and normal conditions.

Our results revealed that the Pielou evenness index in the middle ear was significantly lower in the COME group compared with normal controls. Normal middle ear samples displayed a concentrated distribution on the PCoA plot, reflecting a diverse yet stable microbial community. In contrast, COME samples were more dispersed, with lower alpha diversity, suggesting a potential dominance of specific pathogens in the disease state. The absence of significant differences in other alpha diversity indices suggests that microbial richness was largely preserved, and dysbiosis in COME mainly reflected reduced evenness rather than loss of taxa. Although previous studies have not conducted direct comparisons between control middle ear samples and COME effusions, two studies have demonstrated significantly lower microbial diversity in the nasopharynx of COME patients compared with controls [23, 24]. Additionally, Jervis‐Bardy et al. [9] reported a lower Simpson's index in middle ear fluid compared with the adenoid and nasopharynx, and Sokolovs‐Karijs et al. [25] observed a reduced Pielou index in COME effusions relative to healthy adenoid microbiota. These results collectively support the hypothesis that alterations in specific bacterial populations contribute to microbial dysbiosis in COME.

Previous studies on the microbial composition of the normal middle ear are limited, making it challenging to analyze the role of microbial changes in the pathogenesis of COME. While there has been debate regarding whether the normal middle ear harbors microbial colonization, our findings provide evidence that the healthy middle ear is not a completely sterile environment. Instead, it shares many genera with the nasal cavity and oral cavity, suggesting a potential connection between these anatomical sites. Specifically, our study found that the dominant phyla in the normal middle ear are Proteobacteria, Firmicutes, Bacteroidota, and Actinobacteriota, which is consistent with the findings of Minami et al. [26] and Lee et al. [27].

Several microbial biomarkers for COME, including *Aeromonas*, *Serratia*, and *Lactococcus*, were shared between otic and nasal samples in our study. These genera, which were significantly elevated in the diseased state, exhibited strong correlations in relative abundance between the ear and nasal sites, frequently co‐occurring and demonstrating high predictive value for COME. Given the anatomical proximity of the eustachian tube to the nasal and oral cavities, it has been hypothesized that the nasal cavity may serve as a reservoir for otopathogens, allowing bacteria to ascend the eustachian tube and contribute to the development of COME [28]. This hypothesis is consistent with our findings. Although distinguishing causative changes from consequential ones is challenging, the presence of potential “culprit” microbes implicated in the pathogenesis of COME offers a valuable foundation for future mechanistic investigations. It also highlights the potential for combined nasal and otic therapeutic approaches for COME.

*Aeromonas* is a Gram‐negative rod, with several species known to cause a variety of infectious diseases [29]. It can promote biofilm formation and adhere to biological surfaces via quorum‐sensing systems, making it difficult to eradicate [30]. Additionally, *Aeromonas* harbors inducible chromosomal β‐lactamases, which may confer resistance to antibiotics such as penicillin, ampicillin, and cefazolin [31]. This potential resistance may explain why conventional antibiotic treatments often fail to cure COME.

*Serratia*, a genus of Gram‐negative, facultatively anaerobic, rod‐shaped bacteria, is recognized as an opportunistic pathogen. Among its members, *Serratia marcescens* is particularly notable as a pathogen capable of causing a wide range of clinical diseases, from urinary tract infections to pneumonia. It is now considered an established clinical pathogen, with multi‐antibiotic‐resistant isolates increasingly prevalent [32]. Uddén et al. reported that *Serratia marcescens* was identified in ear discharge and nasopharyngeal swabs from some patients with chronic suppurative otitis media [33]. In our study, certain COME cases showed a high relative abundance of *Serratia* in otic and nasal samples, suggesting its potential involvement in a specific subtype of COME.

*Lactococcus*, a genus of lactic acid bacteria, is generally considered to be of low virulence and is rarely associated with pathogenicity. However, in rare instances, it has been implicated in serious infections such as endocarditis, peritonitis, and intra‐abdominal infections [34]. Notably, *Lactococcus lactis* has also been isolated from ear discharge and nasopharyngeal swabs in some cases with chronic suppurative otitis media [33]. In contrast, in studies of acute otitis media associated with upper respiratory infections, *Lactococcus* was associated with a reduced risk of pneumococcal colonization and otitis media [35]. This discrepancy may reflect stage‐specific ecological adaptations, whereby *Lactococcus* contributes to microbial stability in the healthy or acute phase but expands opportunistically in the chronic inflammatory environment of COME.

To the best of our knowledge, this study is the first to compare the functional composition of microbiota in the middle ear, nasal cavity, and oral cavity under both COME and healthy conditions. While the microbial species composition varied significantly across these anatomical sites, the functional composition remained largely consistent. This observation is in line with previous reports, which have highlighted the presence of core functions essential for microbial survival across different niches [36]. Several pathways were enriched in otic samples from patients with COME. Notably, the two‐component system, a common bacterial signaling mechanism, senses environmental stimuli and stresses to regulate cell motility, biofilm formation, and pathogenesis, aiding bacterial survival and host colonization [37]. The phosphotransferase system, a multicomponent group translocation system absent in eukaryotes, is an attractive target for antimicrobial agents. In addition to its metabolic role, it regulates biofilm formation, antibiotic resistance, and virulence [38, 39]. Cell motility allows bacteria to move toward favorable conditions, aiding colonization and infection [40]. These COME‐enriched mechanisms enable bacteria to sense environmental cues, transport molecules, and coordinate behaviors like motility and surface adherence. The resulting biofilms enhance bacterial resistance to antibiotics and immune responses, playing a key role in COME pathogenesis. These findings may offer new insights into future research targeting interspecies bacterial communication and evaluating the antibiofilm effects of novel compounds for the treatment of otitis media [41, 42].

This study also has several limitations. First, the sample size for the middle ear microbiome of the Control group was limited, as obtaining samples from the control group individuals is inherently challenging. Similar to previous studies [26, 43], middle ear swabs from patients with noninflammatory conditions such as middle ear malformations, otosclerosis, and acoustic neuroma were used to represent controls. However, potential differences between these and samples from individuals without any middle ear pathology cannot be excluded, which may introduce bias. Second, although 16S rRNA sequencing provides high sensitivity for profiling microbial communities, its genus‐level resolution restricts the ability to distinguish between pathogenic and nonpathogenic members within the same genus. Future investigations employing third‐generation sequencing or metagenomic approaches could achieve strain‐level resolution and more precise functional profiling, enabling verification of the current results and identification of potential pathogenic taxa. Third, the absence of virome analysis prevents a comprehensive assessment of viral contributions to COME pathogenesis. Finally, while this study identified several microbial taxa and pathways associated with COME, these biomarkers should be interpreted with caution, as the cross‐sectional design precludes determining whether they represent causal factors or secondary consequences of the disease. Some of these alterations, such as the enrichment of biofilm‐related pathways, may reflect adaptations to the chronic inflammatory environment rather than initiating events. Future mechanistic and longitudinal studies are needed to clarify these causal relationships.

Overall, our results identified potential microbial biomarkers for COME and highlighted the connection between the middle ear and URT microbiota. Future studies are needed to clarify these associations and explore their dynamic interactions. Such efforts will enhance our understanding of COME pathogenesis and facilitate the development of more effective therapeutic strategies.

## 4 Materials and Methods

### 4.1 Participants

A total of 100 patients diagnosed with COME by otolaryngology specialists were enrolled in the Department of Otolaryngology‐Head and Neck Surgery at Beijing Tongren Hospital between January 2022 and March 2024. All patients were scheduled for tympanostomy tube placement. Clinical data, as well as middle ear, oral, and nasal microbiota samples, were collected. Clinical data included disease duration, medical history (AR, CRS, LPRD, snoring, and COVID‐19), smoking and alcohol consumption history, and audiological examination results (pure‐tone audiometry and tympanometry). Patients with cleft palate, immune deficiencies, or nasopharyngeal malignancies who had undergone head and neck radiotherapy or chemotherapy were excluded. In addition, 77   participants without COME were recruited as a control group. Among these, normal middle ear microbiota samples were collected from 25 individuals who underwent middle ear surgery for non‐otitis conditions, including middle ear malformations, traumatic ossicular dislocations, otosclerosis, and acoustic neuroma. Oral and nasal samples were also obtained from all control participants.

All study procedures were approved by the Ethics Review Committee of Beijing Tongren Hospital and registered in the Chinese Clinical Trial Registry (registration number: MR‐1‐24‐056273). Written informed consent was obtained from all adult participants or the parents/guardians of child participants before enrollment.

### 4.2 Sample Collection

Samples were collected using sterile swabs (Copan, Italy). The tongue coating, soft palate, buccal mucosa, and posterior pharyngeal wall mucosa were gently scraped to obtain oral samples. Nasal samples were collected using another sterile swab by gently and repeatedly rotating it against the mucosa of the middle nasal meatus. For patients with COME, effusion was aspirated from the tympanic cavity with sterile suction during surgery for COME and then collected using a sterile swab. For the control group, sterile swabs were gently rotated and carefully wiped on the mucosa near the eustachian tube orifice in the middle ear cavity to collect otic samples during surgery. Throughout the entire collection process, strict precautions were taken to prevent contamination by ensuring that the swabs did not come into direct contact with other body sites, the surrounding environment, or the openings of the sample tubes. Samples were promptly placed on ice and stored at −80°C within 10 min for further processing.

### 4.3 DNA Extraction, Polymerase Chain Reaction Amplification, and 16s rRNA Gene Sequencing

Total genome DNA was extracted using the Cetyltrimethylammonium Bromide (CTAB) method and diluted to 1 ng/µL with sterile water. The V3‐V4 region of the 16S rRNA gene was amplified using specific primers (5’‐CCTAYGGGRBGCASCAG‐3’ and 5’‐GGACTACNNGGGTATCTAAT‐3’) with barcodes. Polymerase chain reaction (PCR) reactions included 15 µL Phusion High‐Fidelity PCR Master Mix (New England Biolabs), 2 µM primers, and 10 ng template DNA, under the conditions of 98°C for 1 min, 30 cycles of 98°C for 10 s, 50°C for 30 s, 72°C for 30 s, and finally 72°C for 5 min. PCR products were verified via 2% agarose gel electrophoresis, pooled in equal density, and purified using the Qiagen Gel Extraction Kit (Qiagen, Germany). Sequencing libraries were prepared with the TruSeq DNA PCR‐Free Kit (Illumina, USA), indexed, and evaluated with the Qubit 2.0 Fluorometer and Agilent 2100 system before sequencing on the Illumina NovaSeq platform to generate 250 bp paired‐end reads.

### 4.4 Bioinformatics Processing and Analysis

Paired‐end reads were assigned to samples based on their barcodes, with barcodes and primer sequences removed during trimming. Analysis of 16S rRNA data was conducted using QIIME2 (version 2023.7). DADA2 was used for denoising, producing an ASV‐level abundance table and microbial feature sequences. The feature sequences were classified against the Silva 138 99% reference database using the sklearn classifier. The abundance table was then collapsed to the genus level.

### 4.5 Statistical Analysis

Group differences were compared using the Wilcoxon test for continuous variables and Fisher's exact test for categorical ones. Bar plots were used to depict the relative abundance of microbiota at the phylum and genus levels for combined group samples. Alpha and beta diversity analyses were performed using the *vegan* package in R. Alpha diversity metrics, including the Shannon, Pielou, Simpson, and Chao1 indices, which reflect the diversity within a single sample, were compared between groups, with higher values of these indices indicating greater diversity. Beta diversity, representing the similarity or dissimilarity between communities, was assessed using Bray–Curtis distances and visualized with PCoA. Group differences in beta diversity were assessed using perMANOVA. Venn diagrams were employed to illustrate the relationships in genus composition and relative abundance across groups. Biomarkers for each group were identified using the LEfSe algorithm, applying thresholds of an LDA score >2 and FDR‐adjusted *p* < 0.05. Microbial community networks were constructed using the *WGCNA* package, with Spearman's rank correlations at the genus level applied under thresholds of FDR‐adjusted *p* < 0.01 and a correlation cutoff of 0.66. Network modularity was analyzed using the *igraph* package and the *cluster_fast_greedy* algorithm. In otic and nasal samples, a 6:4 ratio was used to split the data into training and testing sets. Key biomarkers were used to build random forest predictive models for COME status, with model performance evaluated by receiver operating characteristic (ROC) curves and the AUC. The correlation of abundance between key biomarkers within the same patient was assessed using Spearman's rank correlation. Functional profile predictions based on microbial feature sequences were conducted using the *Tax4Fun2* package, and functional biomarkers were identified using the LEfSe algorithm with an LDA score >3 and FDR‐adjusted *p* < 0.05. All analyses were conducted using R version 4.3.2.

## Author Contributions

Jin Li: Conceptualization, resources, investigation, data curation, writing – original draft; Shenglong Xu: Methodology, formal analysis, data curation, writing – original draft; Qin Gu: Resources, data curation, investigation; Xuan Sun: Resources, data curation; Yahan Zhao: Resources, data curation; Peng Zhang: Conceptualization, methodology, supervision; Yi Li: Conceptualization, resources, investigation, data curation, supervision; yan zhao: conceptualization, resources, investigation, data curation, supervision, writing – review & editing; Luo Zhang: Conceptualization, supervision, writing – review & editing. All authors have read and approved the final manuscript.

## Conflicts of Interest

The authors declare no conflicts of interest.

## Ethics Statement

All study procedures were approved by the Ethics Review Committee of Beijing Tongren Hospital and registered in the Chinese Clinical Trial Registry (registration number: MR‐1‐24‐056273). Written informed consent was obtained from all adult participants or the parents/guardians of child participants before enrollment.

## Supporting information




**Figure S1**: Alpha and beta diversity in COME and Control groups across sampling sites. (A) Comparison of alpha diversity (Simpson and Chao1 indices) between COME and control groups across otic, nasal, and oral sites. (B) Principal coordinate analysis (PCoA) based on Bray–Curtis distance showing beta diversity patterns for otic, nasal, and oral microbiota. The analysis included *n* = 100 (Otic_COME), 25 (Otic_Control), 96 (Nasal_COME), 77 (Nasal_Control), 100 (Oral_COME), and 76 (Oral_Control) samples.
**Figure S2**: Taxonomic composition of the microbiota in COME and Control groups. (A) Mean relative abundance of the top eight phyla in otic, nasal, and oral samples. (B) Mean relative abundance of the top 12 genera in otic, nasal, and oral samples. (C) Venn diagrams depicting the number of genera identified in Control and COME groups across otic, nasal, and oral sites, with percentages in parentheses representing their contribution to the total read counts. The analysis included *n* = 100 (Otic_COME), 25 (Otic_Control), 96 (Nasal_COME), 77 (Nasal_Control), 100 (Oral_COME), and 76 (Oral_Control) samples.
**Table S1**: Comparison of key biomarkers among different subgroups in otic samples from patients with COME.

## Data Availability

The 16S rRNA sequencing dataset is publicly accessible on the Genome Sequence Archive (GSA). The dataset can be accessed at https://ngdc.cncb.ac.cn/gsa (accession number: CRA032242).
