# Supplementary material for: Dysbiosis and Links of the Middle Ear, Nasal, and Oral Microbiota in Chronic Otitis Media with Effusion
Source: MedComm (2020). 2025 Dec 14;6(12):e70550. doi: 10.1002/mco2.70550 (PMC12703046; doi:10.1002/mco2.70550)
Supplement: Supplementary file 1 — Figure S1: Alpha and beta diversity in COME and Control groups across sampling sites. (A) Comparison of alpha diversity (Simpson and Chao1 indices) between COME and control groups across otic, nasal, and oral sites. (B) Principal coordinate analysis (PCoA) based on Bray–Curtis distance showing beta diversity patterns for otic, nasal, and oral microbiota. The analysis included n = 100 (Otic_COME), 25 (Otic_Control), 96 (Nasal_COME), 77 (Nasal_Control), 100 (Oral_COME), and 76 (Oral_Control) samples. Figure S2: Taxonomic composition of the microbiota in COME and Control groups. (A) Mean relative abundance of the top eight phyla in otic, nasal, and oral samples. (B) Mean relative abundance of the top 12 genera in otic, nasal, and oral samples. (C) Venn diagrams depicting the number of genera identified in Control and COME groups across otic, nasal, and oral sites, with percentages in parentheses representing their contribution to the total read counts. The analysis included n = 100 (Otic_COME), 25 (Otic_Control), 96 (Nasal_COME), 77 (Nasal_Control), 100 (Oral_COME), and 76 (Oral_Control) samples. Table S1: Comparison of key biomarkers among different subgroups in otic samples from patients with COME. [file MCO2-6-e70550-s001.docx]

**Supplementary Information**

**Title:** Dysbiosis and Links of the Middle Ear, Nasal, and Oral Microbiota in Chronic Otitis Media with Effusion

**Running Title:** Microbial Dysbiosis in Chronic Otitis Media with Effusion

**Authors:** Jin Li^#1,2^, Shenglong Xu^#1,2^, Qin Gu^#1,2^, Xuan Sun^1,2^, Yahan Zhao^1,2^, Peng Zhang^3^, Yi Li^*1^, Yan Zhao^*1,2^, Luo Zhang^*1,2,4^

**Affiliations:**

^1^ Department of Otolaryngology, Head and Neck Surgery, Beijing TongRen Hospital, Capital Medical University, Beijing, China

^2^ Beijing Institute of Otolaryngology, Beijing Laboratory of Allergic Diseases, Beijing Key Laboratory of New Medicine and Diagnostic Technology Research for Nasal Disease, Beijing, China

^3^ Beijing Key Laboratory for Genetics of Birth Defects, Beijing Pediatric Research Institute; MOE Key Laboratory of Major Diseases in Children; Rare Disease Center, Beijing Children's Hospital, Capital Medical University, National Center for Children's Health, Beijing, China

^4^ Department of Allergy, Beijing TongRen Hospital, Capital Medical University, Beijing, China

^#^Contributed equally

*** Correspondence:** Yi Li (alinaliyi@163.com), Yan Zhao (zhaoyanray@126.com) and Luo Zhang (dr.luozhang@139.com)

**Figure S1.** Alpha and beta diversity in COME and Healthy groups across sampling sites. (A) Comparison of alpha diversity (Simpson and Chao1 indices) between COME and Healthy groups across otic, nasal, and oral sites. (B) Principal coordinate analysis (PCoA) based on Bray–Curtis distance showing beta diversity patterns for otic, nasal, and oral microbiota. The analysis included n = 100 (Otic_COME), 25 (Otic_Healthy), 96 (Nasal_COME), 77 (Nasal_Healthy), 100 (Oral_COME), and 76 (Oral_Healthy) samples.


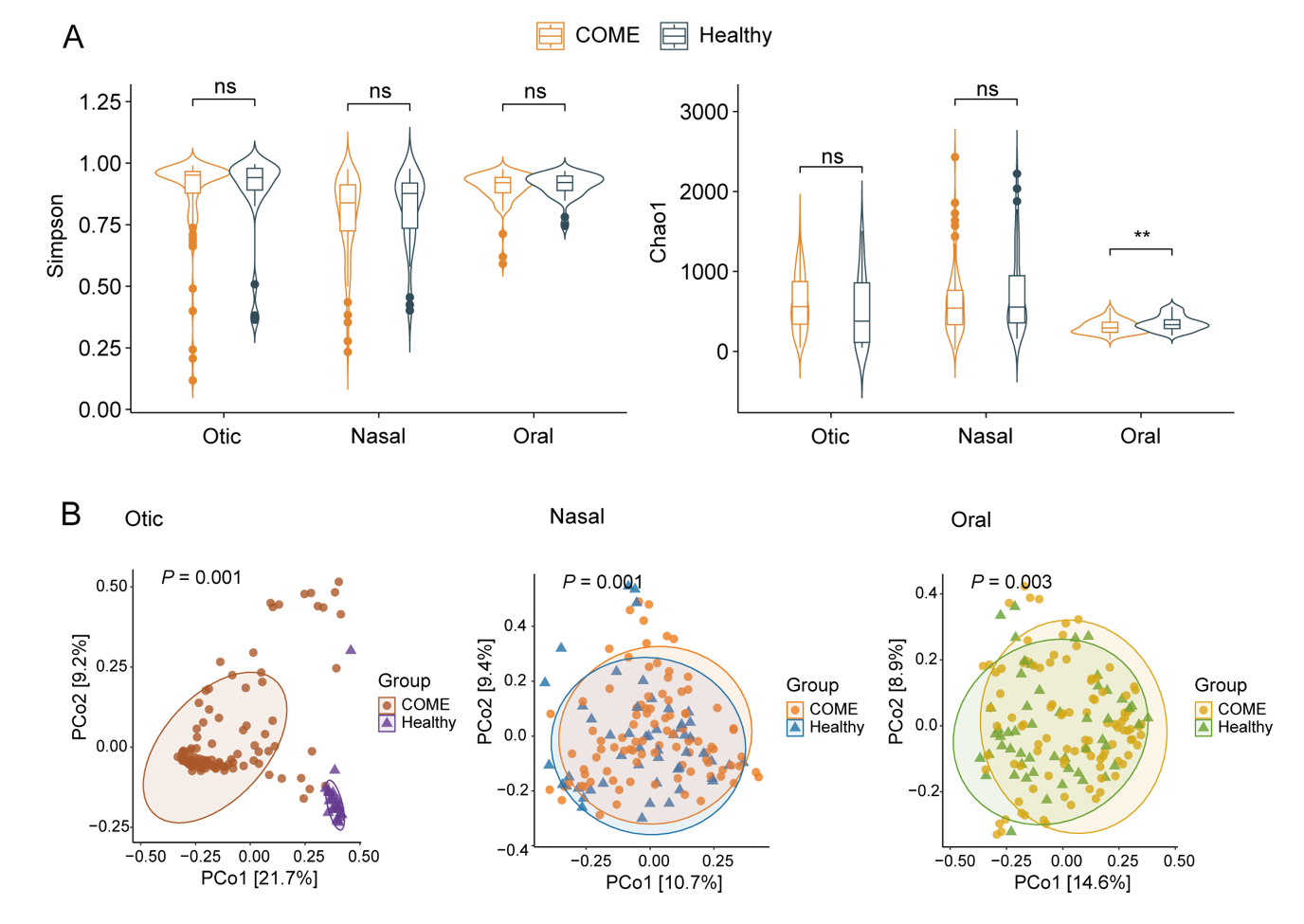


**Figure S2.** Taxonomic composition of the microbiota in COME and Healthy groups. (A) Mean relative abundance of the top 8 phyla in otic, nasal, and oral samples. (B) Mean relative abundance of the top 12 genera in otic, nasal, and oral samples. (C) Venn diagrams depicting the number of genera identified in Healthy and COME groups across otic, nasal, and oral sites, with percentages in parentheses representing their contribution to the total read counts. The analysis included n = 100 (Otic_COME), 25 (Otic_Healthy), 96 (Nasal_COME), 77 (Nasal_Healthy), 100 (Oral_COME), and 76 (Oral_Healthy) samples.


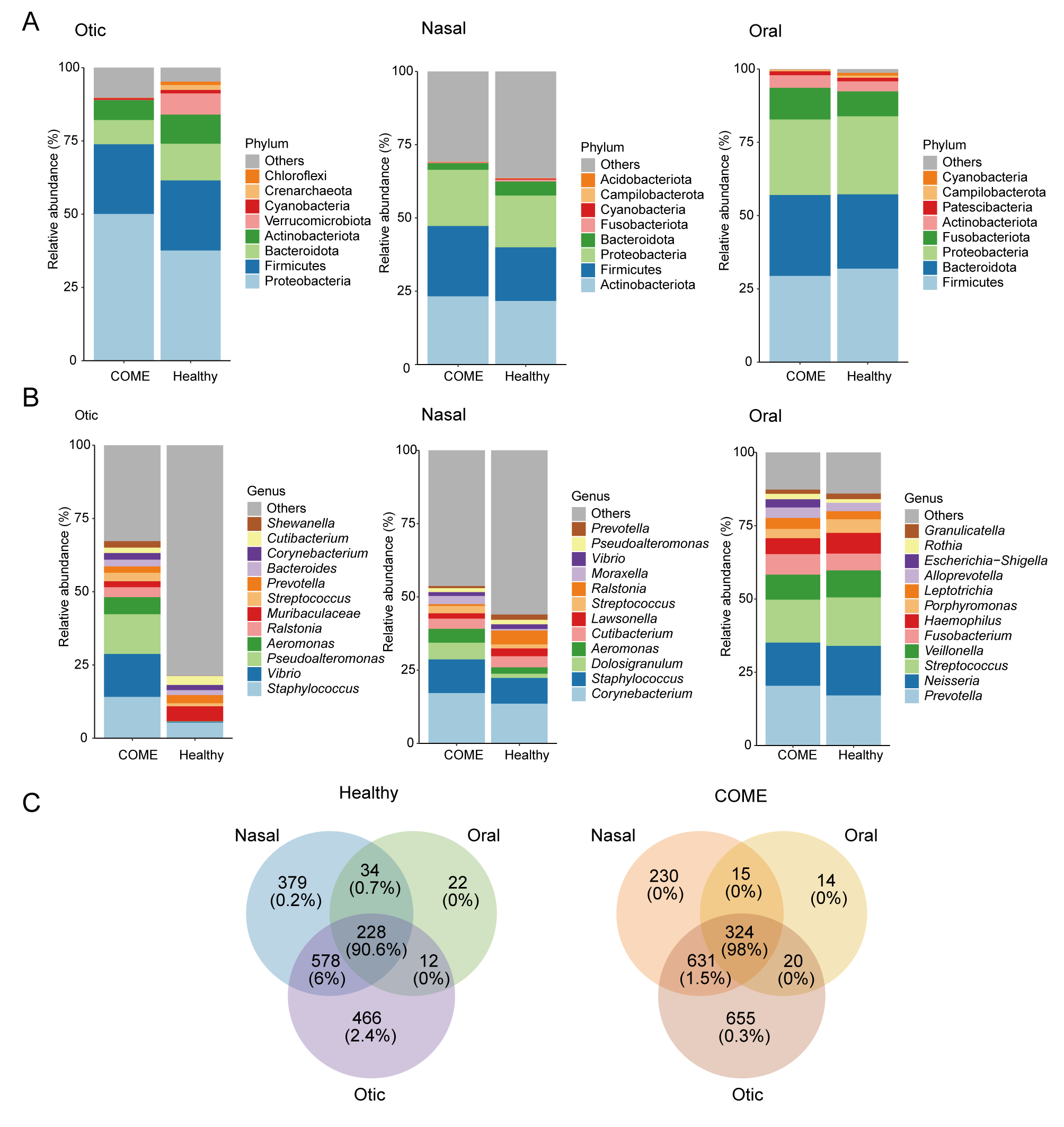


**Table S1.** Comparison of key biomarkers among different subgroups in otic samples from patients with COME.

|  | *g__Aeromonas* | | *g__Serratia* | | *g__Lactococcus* | |
| --- | --- | --- | --- | --- | --- | --- |
|  | Mean±SE | *P*-value | Mean±SE | *P*-value | Mean±SE | *P*-value |
| AR |  |  |  |  |  |  |
| No | 0.057±0.004 | 0.742 | 0.003±0.002 | 0.928 | 0.002±0.000 | 0.742 |
| Yes | 0.065±0.009 |  | 0.001±0.000 |  | 0.001±0.000 |  |
| CRS |  |  |  |  |  |  |
| No | 0.060±0.004 | 0.644 | 0.003±0.002 | 0.644 | 0.002±0.000 | 0.644 |
| Yes | 0.056±0.006 |  | 0.001±0.000 |  | 0.001±0.000 |  |
| LPRD |  |  |  |  |  |  |
| No | 0.058±0.004 | 0.903 | 0.001±0.000 | 0.968 | 0.002±0.000 | 0.918 |
| Yes | 0.062±0.008 |  | 0.007±0.006 |  | 0.001±0.000 |  |
| Snoring |  |  |  |  |  |  |
| No | 0.059±0.004 | 0.852 | 0.003±0.002 | 0.762 | 0.002±0.000 | 0.834 |
| Yes | 0.057±0.006 |  | 0.001±0.000 |  | 0.001±0.000 |  |
| COVID-19 History | |  |  |  |  |  |
| No | 0.054±0.004 | 0.683 | 0.002±0.002 | 0.683 | 0.001±0.000 | 0.738 |
| Yes | 0.071±0.005 |  | 0.002±0.000 |  | 0.002±0.001 |  |
| Tobacco consumption | |  |  |  |  |  |
| No | 0.061±0.004 | 0.736 | 0.003±0.002 | 0.961 | 0.001±0.000 | 0.736 |
| Yes | 0.051±0.008 |  | 0.001±0.000 |  | 0.002±0.001 |  |
| Alcohol consumption | |  |  |  |  |  |
| No | 0.059±0.004 | 0.859 | 0.001±0.000 | 0.808 | 0.001±0.000 | 0.706 |
| Yes | 0.056±0.007 |  | 0.005±0.004 |  | 0.002±0.001 |  |

Group differences were compared using the Wilcoxon test. AR, allergic rhinitis; CRS, chronic rhinosinusitis; LPRD, Laryngopharyngeal reflux disease.
